# Is posttraumatic stress disorder specific to drug‐resistant epilepsy or a common feature of chronic disease? A comparative study with atrial fibrillation and type 1 diabetes

**DOI:** 10.1002/epi.70113

**Published:** 2026-01-24

**Authors:** Lisa‐Dounia Soncin, Capucine Rodet, Mina Aboulmakarim, Bernard Giusiano, Marie Arthuis, Jean‐Claude Deharo, Patrice Darmon, Olivia Ronsin, Sylvane Faure, Fabrice Bartolomei

**Affiliations:** ^1^ Laboratoire d'Anthropologie et de Psychologie Cliniques, Cognitives et Sociales Université Côte d'Azur Nice France; ^2^ Institut de Neurosciences des Systèmes INSERM, Aix Marseille Université Marseille France; ^3^ Epileptology and Cerebral Rhythmology APHM, Hôpital de la Timone Marseille France; ^4^ Service de Cardiologie, Rythmologie APHM, Hôpital de la Timone Marseille France; ^5^ Endocrinologie, Diabète, Maladies Métaboliques APHM, Hôpital de la Conception Marseille France

**Keywords:** chronic disease, emotion regulation, epilepsy, psychiatric comorbidities, psychoepileptogenesis, PTSD, trauma exposure

## Abstract

**Objective:**

Posttraumatic stress disorder (PTSD) is more prevalent in epilepsy than in the general population. However, it remains unclear whether this association is specific to epilepsy or a broader consequence of experiencing unpredictable acute episodes within chronic diseases. This study aimed to (1) compare PTSD prevalence and severity in patients with drug‐resistant epilepsy (DRE) and patients with other chronic episodic diseases, namely, type 1 diabetes (T1D) and drug‐resistant atrial fibrillation (AF); and (2) examine psychiatric comorbidities, emotion regulation, and quality of life across groups.

**Methods:**

A total of 122 patients (64 with DRE; 58 with chronic diseases: 30 with AF, 28 with T1D) completed questionnaires assessing PTSD symptoms, trauma exposure, anxiety, depression, dissociation, emotion regulation, and quality of life. Statistical analyses included χ^2^ tests, *t*‐tests, analyses of covariance controlling for age, and correlation analyses.

**Results:**

PTSD was significantly more prevalent in the DRE group (34.94%) than in the chronic disease group (12.07%; χ^2^ = 9.5, *p* = .004). DRE patients also reported significantly more repeated trauma exposures (54.69% vs. 27.59%, *p* = .004). Compared to other groups, DRE patients showed higher anxiety (*p* = .002), depression (*p* = .002), and dissociation (*p* < .001), poorer emotion regulation (*p* = .003), and lower quality of life (*p* < .001).

**Significance:**

Our findings suggest that the psychiatric burden observed in DRE cannot be solely attributed to chronic episodic disease status. Instead, it may reflect the impact of early and repeated traumatic exposures and their interaction with the neurobiological underpinnings of epilepsy. The strong association between complex trauma, psychiatric comorbidities, and poor quality of life supports the hypothesis of psychoepileptogenesis and calls for integrated trauma‐informed care in epilepsy management.

**Key points**

- The psychiatric burden observed in drug‐resistant epilepsy cannot be explained by chronic disease status alone.
- PTSD is more prevalent and more severe in drug‐resistant epilepsy than in atrial fibrillation or type 1 diabetes.
- Drug‐resistant epilepsy is associated with higher anxiety, depression, dissociation, and emotion regulation deficits.
- Psychiatric comorbidities strongly contribute to reduced quality of life in drug‐resistant epilepsy.
- These findings highlight the critical need to address trauma and PTSD in the care of drug‐resistant epilepsy.

## 1 INTRODUCTION

Psychiatric comorbidities are common in drug‐resistant epilepsy (DRE), with anxiety and depression being the most frequently reported.^1^, ^2^ Recently, posttraumatic stress disorder (PTSD) has emerged as another significant comorbidity in this population. Studies show that PTSD is more prevalent in people with DRE than in healthy controls (24% vs. 7%),^3^, ^4^, ^5^ suggesting that, beyond the neurological disorder, epilepsy is associated with several psychiatric conditions.

Epilepsy appears to be closely linked to both life‐related trauma and the traumatic nature of seizures themselves. Seizures can represent unpredictable, threatening, and sometimes stigmatizing experiences. Altogether, this suggests that seizure may contribute directly to the development of PTSD symptoms.^5^, ^6^, ^7^, ^8^

Recent studies have shown a higher rate of traumatic life experiences and PTSD symptoms in DRE patients than in healthy controls.^3^, ^4^, ^5^ However, whether this vulnerability is specific to epilepsy or reflects the impact of living with a chronic disease (CD) prone to acute episodes remains unclear.

PTSD is a psychiatric condition that can develop after exposure to actual or threatened death, serious injury, or sexual violence, characterized by intrusive memories, avoidance, negative mood, and hyperarousal.^9^ It has also been shown that PTSD in DRE presents with a specific clinical profile, closely associated with the seizure dynamics: avoidance behaviors related to seizures, anticipatory anxiety, hypervigilance toward seizures, and reexperiencing symptoms during the ictal period, among others.^5^

PTSD is also increasingly recognized in chronic illnesses other than DRE. Studies suggest that individuals with chronic somatic conditions, particularly when characterized by episodic manifestations such as diabetes or cardiovascular disease, may develop PTSD as a result of living with the disease itself.^10^, ^11^, ^12^, ^13^, ^14^ Conversely, PTSD is associated with an increased risk of developing chronic conditions through dysregulation of stress systems like the hypothalamic–pituitary–adrenal (HPA) axis and maladaptive behaviors.^11^, ^14^, ^15^ The important question of the difference between cause and effect is beyond the scope of this paper. Patients with type 1 diabetes (T1D) and those with atrial fibrillation (AF) show PTSD symptoms related to acute disease episodes, reactive emotional distress, and the need for continuous self‐management.^10^, ^16^, ^17^, ^18^, ^19^, ^20^ Moreover, AF and T1D share important characteristics with epilepsy compared to other CDs, as acute episodes characterize them. In this context, they are particularly relevant for comparison with DRE.

In this context, we developed a study to better document whether and to what extent the elevated prevalence of PTSD observed in DRE is specific to this condition in comparison to other frequent CDs.

The current study pursued two objectives:

1. The first aim was to compare the prevalence, type of traumatic exposure, and severity of PTSD symptoms in patients with DRE versus those with other chronic episodic diseases, T1D and drug‐resistant AF, to determine whether and how PTSD is related explicitly to epilepsy or more broadly associated with CD that may involve acute symptomatic episodes.
2. The second objective was to explore the impact on quality of life and the psychological and psychiatric profiles (anxiety, depression, dissociation, and emotion regulation) across these two groups (DRE vs. CDs).

We hypothesized that although PTSD is common across various CDs, it may be more pronounced or particularly marked in DRE.

## 2 MATERIALS AND METHODS

### 2.1 Patients

The SPIRALE study (Symptoms of Post‐traumatic Stress in Adult Drug‐Resistant Epilepsies; NCT04749901) is a prospective, open‐label, single‐center, noninterventional study conducted between February 2021 and June 2023 across three departments of Marseille University Hospitals (Assistance Publique–Hôpitaux de Marseille). Adult patients were enrolled during routine medical care and consultations. Patients with DRE, drug‐resistant AF, and T1D were recruited by their attending physicians from the Epileptology and Cerebral Rhythmology Department (Timone Hospital), the Cardiology and Rhythmology Department (Timone Hospital), and the Endocrinology, Diabetes, and Metabolic Diseases Department (Conception Hospital), respectively. To address the study objectives, DRE patients were compared with patients living with other chronic episodic conditions, including AF and T1D (CD group), allowing a focused comparison between DRE and other chronic conditions characterized by intermittent acute episodes.

Patients aged 18–70 years were recruited during routine care from specialized departments. Diagnoses were established by the referring clinicians using standard clinical and paraclinical criteria. Exclusion criteria included current or past psychiatric disorders and cognitive impairment. For DRE, inclusion required a confirmed diagnosis of DRE per International League Against Epilepsy criteria.^21^ Patients with psychogenic nonepileptic seizures without epilepsy were excluded. AF was defined as persistent or recurrent despite antiarrhythmic treatment and according to 2024 ESC Guidelines,^22^ and T1D was diagnosed based on clinical and biological criteria.^23^ The frequency of acute episodes (seizures, glycemic events, and AF episodes) was systematically recorded across all groups as an index of disease severity.

All participants provided informed consent. The study was approved by the French ethics committee Comité de Protection des Personnes SUD‐EST 1 (ID‐RCB: 2020‐A01829‐30). Eligible patients were informed about the study by their referring physician during their routine consultation. After receiving a clear explanation of the research and its procedures, patients signed a nonopposition consent form. Demographic, lifestyle, and clinical data were collected at baseline.

Each participant completed a series of questionnaires independently after receiving detailed instructions. This was followed by a semistructured clinical interview with the hospital psychologist involved in the project.

### 2.2 Psychoemotional assessment and independent variables

#### 2.2.1 Questionnaires

Several validated self‐report questionnaires were administered to assess key well‐being, psychiatric, and emotional dimensions:

- *Quality of Life:* Measured using the French version of the Short Form 12 (SF‐12), a short form derived from the SF‐36, assessing eight physical and psychological dimensions. Higher scores indicate better quality of life.^24^, ^25^
- *Emotion Regulation:* Evaluated using the Difficulties in Emotion Regulation Scale–French version (DERS‐F), based on the original 36‐item scale. Higher scores indicate greater difficulties in emotion regulation.^26^, ^27^
- *Anxiety:* The Beck Anxiety Inventory (BAI; French version) assesses anxiety symptoms across 21 items on a 4‐point Likert scale. Total scores categorize anxiety as minimal (0–7), mild (8–15), moderate (16–25), or severe (26–63).^28^, ^29^
- *Depression:* The Beck Depression Inventory‐Fast Screen–French version (BDI‐FS‐Fr) assesses depressive symptoms over the past 2 weeks using seven items. A score of ≥4 is considered clinically significant.^30^, ^31^
- *Dissociation:* Assessed using the French version of the Dissociative Experience Scale (DES), comprising 28 items scored on a 10‐point Likert scale. A score of ≥25 indicates a high risk of dissociative symptoms^32^, ^33^
- *PTSD:* Measured using the French version of the PTSD Checklist for DSM‐5 (PCL‐5). A score of >31 is considered indicative of probable PTSD.^34^

#### 2.2.2 Semistructured psychological interview

After completing the questionnaires, patients underwent a focused semistructured interview conducted by a trained psychologist. The interview collected qualitative data on traumatic experiences, both related and unrelated to their medical condition, based on Diagnostic and Statistical Manual of Mental Disorders, 5th edition (DSM‐5) PTSD exposure criteria. PTSD symptoms were further evaluated using the Clinician‐Administered PTSD Scale for DSM‐5 (CAPS‐5). During the semistructured interview, patients were asked to describe traumatic events in two categories: (1) disease‐related events (e.g., seizures, hypoglycemia, AF episodes) and (2) non‐disease‐related lifetime trauma (e.g., accidents, violence, neglect). In addition, the interview collected information on the nature of trauma exposure, whether it involved a single or repeated event, as well as the life stage during which the trauma occurred (childhood vs. adulthood). PTSD symptoms were assessed using the PCL‐5, and diagnostic validation was supported by a semistructured clinical interview inspired by the CAPS‐5 protocol, although the CAPS‐5 itself was not formally administered.

### 2.3 Statistical analysis

The main outcome variables were derived from the questionnaires assessing quality of life, emotion regulation, anxiety, depression, dissociation, and PTSD symptoms as well as from data on traumatic exposure (classified as exposed or unexposed, and further distinguished as single, repeated, or disease‐related exposure). Data distribution was examined using the Shapiro–Wilk test, and group comparisons were conducted using independent samples *t*‐tests or nonparametric Mann–Whitney *U*‐tests when appropriate. We compared the type of exposure between the two groups using binary coding (0 = not exposed and 1 = exposed). To control for potential confounding by age, a multivariate analysis of covariance (MANCOVA) was conducted with age as a covariate. Follow‐up analyses of covariance (ANCOVAs) were performed for each outcome variable to determine whether group differences remained significant after adjusting for age. Although the primary analyses contrasted patients with DRE with those with other CDs, the Supporting Information Table provides descriptive data for each diagnostic group (DRE, AF, 1TD) to clarify disease‐specific trends.

## 3 RESULTS

The DRE group was significantly younger (mean = 39.80, SD = 12.85) than the CD group (mean = 47.67, SD = 14.22; Table 1). We have controlled for the effect of agein our analysis in Section 3.3. No significant group differences were found for gender distribution or duration of illness (Table 1).

**TABLE 1:** Personal, sociodemographic, and clinical descriptive characteristics of the two experimental groups.

Characteristic	DRE, *n* = 64	CD, *n* = 58	*p*
Age, years	Mean = 39.80, SD = 12.85	Mean = 47.67, SD = 14.22	*p* = .003*
Time since onset of disease, years	Mean = 13.53, SD = 10.30	Mean = 15.30, SD = 9.70	*p* = .410
Sex ratio, F/M	24/40 (37.5%/62.5%)	24/34 (41.4%/58.6%)	χ^2^ = .103 (*df* = 1) *p* = .748
Type of epilepsy			
Generalized epilepsy	4 (6.25%)	–	
Temporal epilepsy	50 (78.12%)	–	
Posterior epilepsy	4 (6.25%)	–	
Frontal epilepsy	2 (3.125%)	–	
Others	4 (6.25%)	–	
Laterality			
Left focal laterality	29 (45.31%)	–	
Right focal laterality	25 (39%)	–	
Bilateral laterality	4 (6.24%)	–	
Laterality unknown	6 (9.37%)	–	

*Note*: Values are presented as mean with SD or as counts. Group comparisons were performed using *t*‐tests for continuous variables and χ^2^ tests for categorical variables. Probability values reflect significance of group comparison.

Abbreviations: CD, chronic disease; DRE, drug‐resistant epilepsy; F, female; M, male.

* Statistically significant difference between groups (*p* < .05).

### 3.1 Traumatic exposure and PTSD

To assess whether PTSD is more frequent/severe in DRE patients with epilepsy than other CD patients, we first compared traumatic exposure between groups. Although patients with DRE reported higher rates of traumatic exposure than those with other CDs, this difference did not reach statistical significance. However, the nature of the exposure differed; DRE patients reported significantly more frequently repeated direct traumatic events than the other group (Table 2).

**TABLE 2:** Descriptive data on traumatic exposure collected during the psychological interview.

	DRE, *n* = 64		CD, *n* = 58		*p*
Exposed	58	90.63%	44	75.86%	.050
Unexposed	6	9.38%	14	24.14%	.050
Single direct exposure	22	34.38%	26	44.83%	.313
Repeated direct exposure	35	54.69%	16	27.59%	.004*
Indirect exposure	1	1.56%	2	3.45%	.604
Traumatic symptoms episode/seizure	44	68.75%	34	58.62%	.329
Potential PTSD (interview and PCL‐5)	23	35.94%	7	12.07%	.004*

*Note*: All variables are coded binarily: 0 = present, 1 = not present. Statistical comparisons were performed using χ^2^ test.

Abbreviations: CD, chronic disease; DRE, drug‐resistant epilepsy; PCL‐5, PTSD Checklist for DSM‐5; PTSD, posttraumatic stress disorder.

* Statistically significant difference between groups (*p* < .05).

In terms of PTSD symptoms, patients with DRE reported significantly higher levels of distress on the PCL‐5 scale compared to patients with CDs. This difference was statistically significant (*t*_120_ = 3.81, *p* < .001), indicating a more severe expression of PTSD in the epilepsy group. This result is confirmed by a higher rate of potential PTSD in DRE than in CD (Table 2).

### 3.2 Psychiatric comorbidities

**FIGURE 1:**
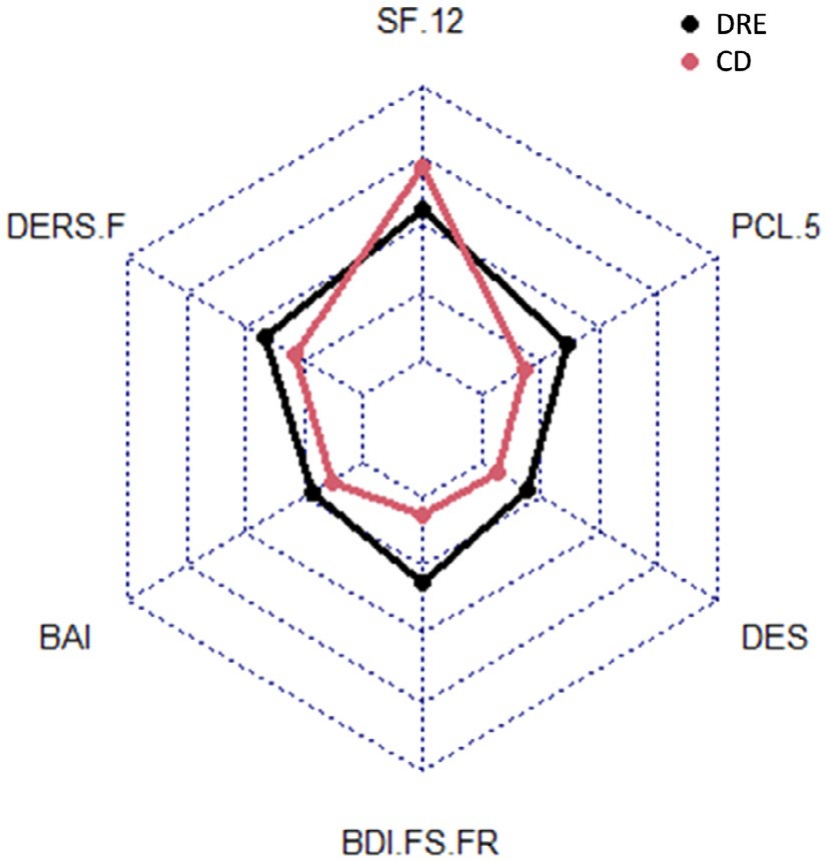
Radar figure showing a comparison of the main variables between patients with drug‐resistant epilepsy (DRE) and other chronic diseases (CD; atrial fibrillation and type 1 diabetes). BAI, Beck Anxiety Inventory; BDI.FS.FR, Beck Depression Inventory‐Fast Screen–French version; DERS.F, Difficulties in Emotion Regulation Scale–French version; DES, Dissociative Experience Scale; PCL.5, PTSD Checklist for DSM‐5; SF.12, Short Form 12.

To characterize the emotional and psychiatric profile of the patients, we compared all the questionnaires' scores between the two groups. The DRE group exhibited significantly higher levels of anxiety (*t*_120_ = 3.23, *p* = .002) and depression (*t*_120_ = 3.10, *p* = .002), as well as more pronounced dissociative symptoms (*t*_120_ = 4.47, *p* < .001), suggesting a more complex psychological burden. In addition, patients with DRE reported greater difficulties in emotion regulation (*t*_120_ = 3.04, *p* = .003) and significantly lower perceived quality of life (*t*_120_ = −3.39, *p* < .001), pointing to a broader negative impact on general well‐being (Figure 1, Tables 3 and 4).

**TABLE 3:** Descriptive statistics and group comparisons for psychological and psychiatric measures.

Measure	DRE, *n* = 64	CD, *n* = 58	*p*
SF‐12	Mean = 37.1, SD = 7.96	Mean = 41.6, SD = 6.42	<.001*
	Min =17, Max = 53	Min =21, Max = 53	
DERS‐F	Mean = 91.5, SD = 23.5	Mean = 79, SD = 21.5	.003*
	Min = 17, Max = 50	Min =44, Max = 148	
BAI	Mean = 16.9, SD = 14.4	Mean = 10, SD = 7.46	.002*
	Min = 0, Max = 59	Min =0, Max = 35	
BDI‐FS‐FR	Mean = 4.95, SD = 4.18	Mean = 2.78, SD = 3.51	.002*
	Min = 0, Max = 16	Min =0, Max = 15	
DES	Mean = 20.5: SD = 18.5	Mean = 8.53, SD = 9.13	<.001*
	Min = 0, Max = 73.6	Min =.357, Max = 49.3	
PCL‐5	Mean = 24.9, SD = 16.7	Mean = 14.2, SD = 14	<.001*
	Min = 0, Max = 61	Min =0, Max = 60	

*Note*: Mean, SD, Min, and Max values for each group. Probability values reflect results from independent samples *t*‐tests.

Abbreviations: BAI, Beck Anxiety Inventory; BDI‐FS‐FR, Beck Depression Inventory‐Fast Screen–French version; CD, chronic disease; DERSF‐F, Difficulties in Emotion Regulation Scale–French version; DES, Dissociative Experience Scale; DRE, drug‐resistant epilepsy; Max, maximum; Min, minimum; PCL‐5, PTSD Checklist for DSM‐5; SF‐12, Short Form 12.

* Statistically significant difference (*p* < .05).

**TABLE 4:** Number of patients per group above the diagnostic thresholds of the clinical scales.

Scale	DRE, *n* = 64		CD, *n* = 58		*p*
BAI					.008*
Minor	17	26.56%	26	44.83%	
Mild	23	35.94%	21	36.21%	
Moderate	12	18.75%	7	12.07%	
Severe	12	18.75%	4	6.90%	
BDI‐FS‐Fr	33	51.56%	15	25.86%	.002*
DES	19	29.69%	3	5.17%	<.001*
PCL‐5	23	35.94%	7	12.07%	<.001*

*Note*: Cutoff scores: PCL‐5 > 31 (posttraumatic stress disorder); DES > 25 (dissociation); BDI‐FS‐Fr > 4 (depression); BAI: mild anxiety ≥ 8, moderate ≥ 16, severe ≥ 26. Probability values are from independent samples *t*‐tests.

Abbreviations: BAI, Beck Anxiety Inventory; BDI‐FS‐Fr, Beck Depression Inventory‐Fast Screen–French version; CD, chronic disease; DES, Dissociative Experience Scale; DRE, drug‐resistant epilepsy; PCL‐5, PTSD Checklist for DSM‐5.

* indicates statistically significant difference (*p* < .05).

### 3.3 Controlling for the effect of age

Patients in the CD group were significantly older than those with DRE (*t*_120_ = 3.05, *p* = .003). Age was significantly correlated with all main psychological variables—emotion regulation, anxiety, depression, dissociation, and PTSD symptoms (all *r* > |.390|, all *p* < .002)—except for quality of life (*r* = .173, *p* = .056).

To control for this potential confounding factor, we conducted a MANCOVA with group (DRE vs. CD) as the fixed factor and age as a covariate, examining the following dependent variables together: BAI (anxiety), BDI‐FS (depression), DES (dissociation), PCL‐5 (PTSD symptoms), DERS‐F (emotion regulation), and SF‐12 (quality of life). The analysis revealed a significant multivariate effect of group after adjusting for age (Wilks lambda = .820, *F*_6, 114_ = 4.18, *p* < .001).

We then conducted follow‐up ANCOVAs for each outcome separately using the following model: Outcome Variable ~ Group + Age.

Each ANCOVA confirmed significant group effects on the corresponding psychological measures, indicating that the observed differences between groups were not solely attributable to age.

Complete ANCOVA results including *F*‐values, degrees of freedom, *p*‐values, and partial η^2^ squared values are reported in Table S4.

## 4 DISCUSSION

This study is, to our knowledge, the first to compare prevalence, type of traumatic exposure, and severity of PTSD symptoms in patients with DRE versus those with other chronic episodic diseases (CD): T1D and drug‐resistant AF. We also explore the impact on quality of life as well as psychological and psychiatric profiles (anxiety, depression, dissociation, and emotion regulation) across these two groups (DRE vs. CD).

The primary aim was to determine whether PTSD and its psychiatric correlates are specific to epilepsy or rather reflect a more general consequence of living with a CD that may involve unpredictable acute episodes.

### 4.1 PTSD prevalence and exposure profiles

Our findings indicate that patients with DRE exhibit a significantly higher prevalence of PTSD, as evidenced by the number of patients showing scores above the clinical threshold (as measured by the PCL‐5) compared to those with other CDs (35.9% vs. 12.1%). Although these results are consistent with the literature, the rate observed in our DRE group is notably higher than the prevalence estimates reported in two recent meta‐analyses, which range from 7% to 18%.^3^, ^4^ This discrepancy may stem from methodological differences, particularly the previous meta‐analyses including all forms of epilepsy without distinguishing drug‐resistant cases and using heterogeneous tools for PTSD assessment. Furthermore, participants in our study were recruited from a national reference center for complex and rare epilepsies, implying that the sample likely included patients with more severe and refractory forms of the disorder. These patients may have experienced a longer and more burdensome disease course, including multiple treatment failures, which could increase their vulnerability to developing PTSD and other psychiatric comorbidities.

Regarding trauma exposure frequency and typology, the type of trauma exposure was differently distributed across groups. Regarding frequency of trauma exposure and its typology, our study highlights two results. First, total trauma exposure rates were not significantly different. Second, DRE patients reported more repeated trauma exposures, consistent with complex trauma typology.^35^, ^36^ Specifically, 54.6% of DRE patients reported repeated exposures, compared to 27.6% in the CD group.

Lifetime exposure to traumatic events is estimated at 70.4% worldwide and 72.2% in France, whereas PTSD prevalence stands at around 5.6%.^37^, ^38^ In epilepsy, PTSD prevalence has been estimated between 7% and 18%, with a threefold increased risk relative to the general population.^3^, ^4^ For CDs, a recent study estimated PTSD prevalence at 12.7% in a large cohort.^39^

Psychiatric comorbidities like anxiety and mood disorders are well documented in epilepsy, occurring at higher rates than in the general population.^1^, ^2^ It has been suggested that trauma history, particularly in childhood, and PTSD may help explain this increased burden in DRE beyond the experience of chronic illness itself.^5^, ^40^ Our study is critical in testing this hypothesis by comparing DRE with other chronic episodic diseases that entail similar stressors (e.g., treatment burden, medical monitoring, hospitalization).

Importantly, we selected comparator conditions that, like epilepsy, involve “crises” (e.g., hypoglycemia, hyperglycemia, AF episodes), which demand high somatic vigilance and can foster anticipatory anxiety. Our results suggest that acute episodes within chronic medical conditions alone do not account for the elevated psychiatric comorbidities observed in DRE. Rather, previous trauma and PTSD symptoms are likely to contribute more substantially.

To our knowledge, data on trauma typology (simple vs. complex) are lacking for CDs. However, in epilepsy, clinical observations suggest that repeated seizures may perpetuate a cycle of exposure, fostering PTSD patterns consistent with complex trauma.^5^ Thus, complex trauma may be a specific risk factor in DRE and could explain the increased PTSD burden in this group. Complex trauma has been linked to higher risk and more severe, persistent PTSD.^35^, ^36^

Regarding the types of traumatic exposure, 54.7% of DRE patients reported repeated direct trauma, compared to 27.6% in the CD group. Single trauma exposure was reported by 34.4% of DRE patients versus 44.8% in CD; indirect trauma was rare in both groups (1.6% in DRE, 3.5% in CD). Furthermore, 68.8% of DRE patients reported trauma‐related symptoms during episodes or seizures, compared to 58.6% in CD, suggesting that trauma linked to disease‐specific symptomatology, particularly seizures, may play a central role in PTSD development among DRE patients.

Although the traumatic event initially triggering PTSD is not always seizure‐related, our previous work has shown that PTSD symptomatology such as hypervigilance, avoidance, and dissociation can become strongly associated with seizure dynamics. This process, which we previously described as “seizure traumatogenesis,” suggests that the seizures themselves may progressively acquire a traumatic quality. As such, even when PTSD originates from nonepileptic life events, the inter‐ and peri‐ictal symptom expression may reinforce the traumatic experience of seizures, blurring the distinction between seizure‐related and ‐unrelated PTSD. This supports the hypothesis that the clinical presentation of PTSD in epilepsy has specific features shaped by seizure semiology and CD context.

### 4.2 Psychiatric symptoms, emotion regulation, and quality of life

The second objective was to compare psychiatric symptoms, emotion regulation capacities, and quality of life between the DRE and CD groups. In addition to more trauma exposure and PTSD, DRE patients showed a more severe psychiatric profile, reporting higher levels of anxiety, depression, and dissociation and poorer emotion regulation.

Importantly, DRE patients also reported significantly lower quality of life than those with other CDs. This suggests that the more severe impact on well‐being observed in DRE is not solely due to epilepsy itself, but rather to the psychiatric symptoms, such as PTSD, anxiety, and dissociation, that are closely intertwined with the seizure dynamics. These symptoms likely amplify the anxiogenic and traumatogenic experience of seizures, thereby exacerbating the overall impact of the disease on quality of life. This could explain why epilepsy, particularly in its drug‐resistant form, results in more profound impairment than other chronic conditions.^5^, ^41^

The data highlight a polycomorbid profile in DRE, consistent with a PTSD clinical picture rooted in complex trauma. Dissociative symptoms, more commonly seen in survivors of chronic or multiple trauma, and deficits in emotion regulation, which are frequently associated with psychiatric vulnerability, were more prevalent in the DRE group. These findings align with transdiagnostic models linking impaired emotional regulation with psychopathology.^42^

Trauma and PTSD thus appear more prevalent in DRE than in other chronic illnesses. Moreover, PTSD and its related physiological stress mechanisms have been associated with vulnerability to somatic disease.^14^ The observation that trauma‐related vulnerability is more pronounced in epilepsy might relate to its neurological nature.

Limbic and paralimbic structures involved in stress processing are also implicated in temporal lobe epilepsy, which represented 78.1% of our DRE sample.^5^ This supports the theory of “psychoepileptogenesis,” whereby early trauma and PTSD‐related stress may influence epilepsy onset. In earlier studies, 47%–48.4% of patients reported a temporal link between trauma and epilepsy onset, and 18.5% reported that seizures began within 6 months of a traumatic event.^5^, ^43^ Animal models also support the stress diathesis hypothesis in epileptogenesis.^5^, ^44^, ^45^ These observations, both in humans and animals, remain hypothetical and suggest a plausible mechanism rather than a demonstrated causal link. Furthermore, temporal brain regions, particularly the hippocampus, are known to be highly sensitive to cortisol due to their dense population of glucocorticoid receptors. This sensitivity makes them especially vulnerable to dysregulation of the HPA axis. Chronic stress and elevated cortisol levels may thus enhance neuronal excitability in these regions, potentially contributing to seizure generation. Within this framework, the concept of “psychoepileptogenesis” has not yet been shown to reflect a causal mechanism. It should be understood as a working hypothesis that integrates clinical and experimental observations concerning the relationship between trauma and the onset of epilepsy. Taken together, these results suggest that patients with DRE, particularly those with temporal lobe epilepsy, may be more susceptible to PTSD and psychiatric comorbidity than patients with other chronic conditions prone to acute episodes, due both to trauma history and potentially to temporal lobe involvement in neurological vulnerability.

### 4.3 Limitations

Finally, some limitations should be acknowledged. This is an exploratory study and should be replicated in larger, more diverse samples, including patients with other chronic neurological diseases or other psychiatric conditions. Our sample was small and hospital‐based, limiting generalizability. No physiological measurements were included. Finally, the diseases compared differ substantially in etiology and symptomatology, which may complicate cross‐condition comparisons. In addition, the sample was recruited in a hospital setting specifically, in a national reference center for DRE, and may therefore not fully represent the broader population of patients with epilepsy. Additionally, although we excluded patients with severe psychiatric or cognitive disorders, we did not systematically control for all comorbid psychiatric diagnoses or the use of psychotropic medications, which may have influenced psychological symptomatology and PTSD expression. Future studies with larger, more diverse populations and stricter control of clinical confounders are needed to confirm and expand upon these findings.

## 5 CONCLUSIONS

In summary, this study highlights that PTSD and psychiatric comorbidities are significantly more prevalent and severe in patients with DRE than in those with other chronic episodic conditions. These findings support the hypothesis that complex trauma exposure, more frequent in DRE, plays a key role in the emergence of PTSD symptoms and contributes to a distinct and more debilitating psychiatric profile. Furthermore, the observed association between psychiatric symptoms and reduced quality of life in patients with DRE suggests that emotional and traumatic processes may contribute significantly to the burden of the disease, potentially as much as, or in interaction with, the seizures themselves. These findings highlight the need for systematic screening of trauma history and PTSD symptoms in patients with DRE. Given the strong association between complex trauma, psychiatric burden, and impaired quality of life, early identification and trauma‐informed interventions should be integrated into the clinical management of refractory epilepsy.

## AUTHOR CONTRIBUTIONS

Lisa‐Dounia Soncin, Sylvane Faure, and Fabrice Bartolomei conceptualized the study. Jean‐Claude Deharo, Patrice Darmon, and Fabrice Bartolomei served as the principal investigators and supervised the research protocol. Lisa‐Dounia Soncin, Capucine Rodet, and Mina Aboulmakarim, and Marie Arthuis coordinated patient recruitment and data collection. Lisa‐Dounia Soncin and Bernard Giusiano contributed to data validation and statistical analysis. Fabrice Bartolomei, Jean‐Claude Deharo, Patrice Darmon, Lisa‐Dounia Soncin, and Olivia Ronsin supported clinical inclusion. Sylvane Faure, Fabrice Bartolomei, and Lisa‐Dounia Soncin wrote the first draft of the manuscript. All authors reviewed and approved the final version of the manuscript.

## CONFLICT OF INTEREST STATEMENT

The authors declare no conflict of interest.

## ETHICS APPROVAL AND CONSENT TO PARTICIPATE

This study was conducted in accordance with the principles of the Declaration of Helsinki. The SPIRALE study (Symptoms of Post‐traumatic Stress in Adult Drug‐Resistant Epilepsies; ClinicalTrials.gov identifier: NCT04749901) was approved by the French ethics committee Comité de Protection des Personnes SUD‐EST 1 (ID‐RCB: 2020‐A01829‐30). All participants provided written informed consent prior to enrollment. We confirm that we have read the Journal's position on issues involved in ethical publication and affirm that this report is consistent with those guidelines.

## Supporting information


**DATA S1** Supplementary information.

## Data Availability

The datasets generated and analyzed during the current study are available from the corresponding authors on reasonable request.
