# Supplementary material for: Is posttraumatic stress disorder specific to drug‐resistant epilepsy or a common feature of chronic disease? A comparative study with atrial fibrillation and type 1 diabetes
Source: Epilepsia. 2026 Jan 24;67(5):2440–9. doi: 10.1002/epi.70113 (PMC13179667; doi:10.1002/epi.70113)
Supplement: Supplementary file 1 — DATA S1 Supplementary information. [file EPI-67-2440-s001.docx]

**Supplemental data**

*COVID-19 Disruption Scale*

Given that the study was conducted during the COVID-19 pandemic, a custom 4-item scale was specifically developed by the research team to assess perceived disruption related to the health crisis. This exploratory measure captured three core dimensions: fear of infection, disruption of medical care, and stress due to public health restrictions. Each item was rated on an 11-point Likert scale ranging from 0 (“not at all disturbed”) to 10 (“extremely disturbed”). The total score was used as a secondary control variable in the analyses.

We examined the potential impact of contextual factors, particularly the COVID-19 pandemic, on the main psychological variables. Correlation analyses between the COVID-related disruption scale and core measures—including quality of life, emotion regulation, depression, dissociation, and PTSD symptoms—revealed no significant associations (all *r* < 0.144, all *p*s > 0.113), except for a weak but significant positive correlation with anxiety (BAI, *r* = 0.193, *p* = 0.033). While this suggests that the pandemic context may have slightly increased anxiety levels, the effect was not specific to either group. Indeed, no significant group difference was observed on the COVID-related disruption scores (*t*(120) = 1.76, *p* = 0.081), indicating that any potential increase in anxiety was likely consistent across the entire sample. Therefore, the COVID context does not appear to have confounded the between-group comparisons in this study.

**Supplementary tables**

|  | **DRE**  **(*N*=64)** | | **AF**  **(*N*=30)** | **T1D**  **(*N*=28)** | ***P-*value** |
| --- | --- | --- | --- | --- | --- |
| Age (*M; SD*) | *M*=39,80; *ET*=12,85 | | *M*=55,17; *ET*=12,04 | *M*=39,64; *ET*=15,18 |  |
| Time since onset of disease (in years; *(M; ET)* | | *M*=13,53; *ET*=10,30 | *M*=9,48; *ET*=11,00 | *M*=21,32; *ET*=12,61 | *p=NS* |
| F/M | | 24/40 (37.5%/62,5%) | 6/24 (20%/80%) | 18/10 (64.2%/35.7%) | *p*=0.002;  ***χ^2^***= (12.09 ; *df*=2) |
| **SuppTaple 1**. Personal, socio-demographic, and descriptive clinical information for the three experimental group   \|  \| **DRE (*N*=64)** \| **AF (*N*=30)** \| **T1D (*N*=28)** \| ***p-value*** \| \| --- \| --- \| --- \| --- \| --- \| \| **SF-12** \| *M*=36,1 ; *SD*= 8,34 ;  Min =20 ; Max= 50 \| *M*= 42,8 ; *SD*= 4,56 ;  Min =31 ; Max= 53 \| *M*= 40,3 ; *SD*= 7,83 ;  Min =21 ; Max= 53 \| *0.008* \| \| **DERSF-F** \| *M*= 98,1 ; *SD*= 22,3 ;  Min=63 ; Max= 144 \| *M*= 72 ; *SD*= 17,2 ;  Min =44 ; Max= 112 \| *M*= 86,6 ; *SD*= 23,4 ;  Min =54 ; Max= 148 \| *<0.001** \| \| **BAI** \| *M*=18,1 ; *SD*=15,9 ;  Min=0 ; Max= 59 \| *M*= 8,80 ; *SD*= 5,55 ;  Min =0 ; Max= 22 \| *M*= 11,3 ; *SD*= 9,52 ;  Min =0 ; Max= 35 \| *0.057* \| \| **BDI-FS-FR** \| *M*=5,81 ; *SD*=4,45 ;  Min=0 ; Max=16 \| *M*= 1,87 ; *SD*= 2,60 ;  Min =0 ; Max= 10 \| *M*= 3,15 ; *SD*= 4,11 ;  Min =0 ; Max= 15 \| *<0.001** \| \| **DES** \| *M*=22,5 : *SD*= 19,4 ;  Min=0 ; Max=73,6 \| *M*= 7,95 ; *SD*= 8,33 ;  Min =1,07 ; Max= 39,3 \| *M*= 9,16 ; *SD*= 10 ;  Min =0,357 ; Max= 49,3 \| *<0.001** \| \| **PCL-5** \| *M*=29,1 ; *SD*=17,7 ;  Min=0 ; Max=61 \| *M*= 11,6 ; *SD*= 10,8 ;  Min =0 ; Max= 47 \| *M*= 17 ; *SD*= 16,5 ;  Min =0 ; Max= 60 \| *<0.001** \| \| **SuppTable 2.** *Indices of central tendency and dispersion (mean, standard deviation, min. and max. values) for the three experimental groups.*  SF.12, Quality of life; PCL-5, PTSD; DES, Dissociation; BDI.FS.FR, Depression; BAI, Anxiety; DERS.F, Emotion regulation  ***Significant *P* value \| \| \| \| \|  \|  \| DRE (*N*=60) \| AF (*N*=30) \| T1D (*N*=28) \| *p*-value (***χ^2^***) \| \| --- \| --- \| --- \| --- \| --- \| \| Exposed \| 58 (96.7%) \| 26 (86.7%) \| 18 (64.3%) \| <0.005 \| \| Unexposed \| 2 (3.3%) \| 4 (13.3%) \| 10 (35.7%) \|  \| \| Single direct exposure \| 22 (36.7%) \| 19 (63.3%) \| 7 (25.0%) \| 0.024 \| \| Repeated direct exposure \| 35 (58.3%) \| 7 (23.3%) \| 9 (32.1%) \| <0.005 \| \| Indirect exposure \| 1 (1.7%) \| 0 (0.0%) \| 2 (7.1%) \| 0.194 \| \| Traumatic symptoms episode/crisis \| 44 (73.3%) \| 18 (60.0%) \| 16 (57.1%) \| 0.229 \| \| PTSD diagnosis (interview and PCL-5) \| 23 (38.3%) \| 1 (3.3%) \| 6 (21.4%) \| <0.001 \| \| **SuppTable 3**. Descriptive tables of information relating to traumatic exposure collected during the psychological interview.  ***Significant *P*-value \| \| \| \| \| | | | | |  |

| Outcome Variable | Source | *F* | *df1* | *df2* | *p* | *MS* | *Residual MS* | *η²* |
| --- | --- | --- | --- | --- | --- | --- | --- | --- |
| Global MANCOVA | Group | 7.182 | 8 | 112 | < .001 | - | - | 0.339 |
| Global MANCOVA | Age | 0.904 | 8 | 112 | 0.516 | - | - | 0.061 |
| SF-12 | Group | 11.37 | 1 | 119 | 0.001 | 605.99 | 53.30 | 0.087 |
| SF-12 | Age | 0.06 | 1 | 119 | 0.815 | 2.94 | 53.30 | 0.001 |
| DERS-F | Group | 9.18 | 1 | 119 | 0.003 | 4704.23 | 512.47 | 0.071 |
| DERS-F | Age | 0.47 | 1 | 119 | 0.493 | 242.16 | 512.47 | 0.004 |
| BAI | Group | 10.37 | 1 | 119 | 0.002 | 1438.12 | 138.71 | 0.080 |
| BAI | Age | 0.42 | 1 | 119 | 0.516 | 58.76 | 138.71 | 0.004 |
| BDI-FS-FR | Group | 9.52 | 1 | 119 | 0.003 | 144.24 | 15.15 | 0.074 |
| BDI-FS-FR | Age | 0.01 | 1 | 119 | 0.903 | 0.23 | 15.15 | 0.000 |
| DES | Group | 19.87 | 1 | 119 | < .001 | 4390.43 | 220.99 | 0.143 |
| DES | Age | 0.24 | 1 | 119 | 0.622 | 54.05 | 220.99 | 0.002 |
| PCL-5 | Group | 14.39 | 1 | 119 | < .001 | 3471.86 | 241.22 | 0.108 |
| PCL-5 | Age | 0.04 | 1 | 119 | 0.850 | 8.66 | 241.22 | 0.000 |
| HRCS | Group | 28.52 | 1 | 119 | < .001 | 10025.82 | 351.56 | 0.193 |
| HRCS | Age | 4.07 | 1 | 119 | 0.046 | 1430.22 | 351.56 | 0.033 |
| PTSD-E | Group | 4.49 | 1 | 119 | 0.036 | 1812.51 | 403.45 | 0.036 |
| PTSD-E | Age | 0.06 | 1 | 119 | 0.812 | 22.95 | 403.45 | 0.000 |
| **SuppTable 4.** Multivariate and univariate analyses of covariance (MANCOVA and ANCOVA) were conducted to examine the effect of Group (DRE *vs* CD) and Age on psychological outcomes, adjusting for age as a covariate. Reported statistics include F-values, degrees of freedom (df), p-values, Mean Squares (MS), residual Mean Square (Residual MS), and partial eta squared (*η*²) as an index of effect size. With Bonferoni’s correction for ANCOVA (threshold = 0.0031) | | | | | | | | |
